# Your professionalism is not my professionalism: congruence and variance in the views of medical students and faculty about professionalism

**DOI:** 10.1186/s12909-016-0807-x

**Published:** 2016-11-08

**Authors:** Kamran Sattar, Sue Roff, Sultan Ayoub Meo

**Affiliations:** 1 Department of Medical Education, College of Medicine, King Saud University, Riyadh, Saudi Arabia; 2 Center for Medical Education, University of Dundee, Scotland, UK; 3 Department of Physiology, College of Medicine, King Saud University, P.O. Box 2925, Riyadh, 11461 Saudi Arabia

**Keywords:** Professionalism, Congruence and variance, Medical students

## Abstract

**Background:**

Medical professionalism is an essential aspect of medical education and practice worldwide and it must be adopted according to different social and cultural contexts. We examined the current congruence and variance in the perception of professionalism in undergraduate medical students and faculty members in one medical school in Saudi Arabia.

**Methods:**

The target population was first year to final year medical students of College of Medicine, King Saud University. Out of a total of 1431 students at College of Medicine 750 students (52 %) participated in the study. Fifty faculty members from clinical and non-clinical departments of the College of Medicine were randomly selected for this study and all participated in the study. The respondents recorded their responses through the Bristol online survey system, using a bilingual (English and Arabic) version of the Dundee Polyprofessionalism Inventory I: Academic integrity, which has 34 items.

**Results:**

There are 17 lapses (50 % of the total) in professional behaviour where none of the faculty recommend the ignore sanction while students recommended a variable ignore sanction in a range of 6–29 % for different behaviours. Students and faculty recommended similar sanctions for 5 lapses (14.7 % of the total) in professional behaviours. Furthermore, there is statistically significant two level difference between the sanctions approved by faculty and students in the recommended sanctions for 12 lapses (35 % of the total (*p* < 0.050).

**Conclusions:**

These results raised concerns in relation to the students’ understanding of professionalism. It is therefore, important to enhance their learning around the attributes of medical professionalism.

## Background

Professionalism is defined as a group of attitudes, values, behaviours and interactions that act as the basis of the health professional’s contract with society. Medical students have certain privileges and responsibilities hence, standards of professional behaviour are expected of them [1]. This has initiated a global trend of re-visiting professionalism education and, as a result, professionalism has become an explicit component of medical curricula [2, 3]. Murden et al. [4] have noted that medical educators are encouraged to simultaneously address the importance of monitoring of unprofessional or disruptive behaviours. The role of faculty members in encouraging professional behaviours in students is central. Any educational organization where the faculty is well aware of professional responsibilities can undoubtedly support and provide opportunities for students’ professional behaviours to be promoted. Effective physician role models enable learners to internalize the principles of professionalism so that learners themselves act professionally [5]. It is then essential that both the faculty and the students should maintain professional attributes in their actions, behaviour, and interactions. The educational situations for the majority of the teaching faculty have advanced since their entrance into practice; therefore, assessment of their insights about professionalism is equally essential.

The importance of formulating a framework which can help the medical educators understand and refine their approaches to dealing with unprofessional behavior is important as many Arabian teachers and students feel that professionalism education remains a gap in formal curricula [6]. The Committee of Deans of Medical Schools in the Kingdom of Saudi Arabia established a task force to develop a national competency framework for doctors [3]. It is also noted that efforts are being invested in the development of a method that is suitable to address such issues, e.g. Saudi MEDS competence specification for Saudi medical graduates.

Numerous studies, worldwide, have been published to exhibit the major lapses committed by students while studying in their medical school. Teplitsky et al. [7] and Scheers and Dayton [8] reported that the most common presentations of academic integrity is plagiarism, impersonating a student who is absent from class, forging signatures, obtaining illegal access to examination questions, legitimizing absences by false testimony or bribes, helping others to cheat in examinations, cheating in examinations, and falsifying or fabricating data. Therefore, the present study aimed to attempt through administration of a bilingual (Arabic and English) version of the Dundee Polyprofessionalism Inventory I: Academic integrity [9] to collect and compare the recommended sanctions for professionalism lapses of faculty and students at the College of Medicine, King Saud University.

## Methods

This cross-sectional study was conducted in the Department of Medical Education, College of Medicine of King Saud University, Riyadh, Saudi Arabia during the period Jan-June 2015. The instrument used was the 34-item version of the Dundee Polyprofessionalism Inventory: 1 Academic Integrity which has been used in the UK, Saudi Arabia, Pakistan and Egypt [9].

The target population was first year to final year medical students of College of Medicine, King Saud University. Out of a total of 1431 students at College of Medicine 750 students (52 %) participated in the study. Fifty faculty members from clinical and non-clinical departments of the College of Medicine were randomly selected for this study and all participated in the study.

An anonymous, self-administered, bilingual (Arabic and English) inventory (Dundee Polyprofessionalism Inventory I: Academic Integrity) was used. The survey included consent of the participants. The Institutional Review Board of College of Medicine, King Saud University approved the study. The inventory was sent electronically to all the participants using Bristol Online Survey (BOS). All participants were sent 2 reminder emails (1 week apart) after first request of the survey. Respondents recommended the sanctions (Table 1) for the first time lapses in 34 types of professionalism with no mitigating circumstances by undergraduate medical students. The collected data were entered into Microsoft Excel 2007 and analysed using IBM SPSS Program. A *p*-value of < 0.05 was considered statistically significant.

**Table 1:** List of recommended sanctions [10]

1. Ignore (None)	
2. Reprimand (verbal warning)	
3. Reprimand (written warning)	
4. Reprimand, plus mandatory counselling	
5. Reprimand, counselling, extra work assignment	
6. Failure of specific class/remedial work to gain credit	
7. Failure of specific year (repetition allowed)	
8. Expulsion from college (readmission after one year possible)	
9. Expulsion from college (no chance for readmission)	
10. Report to a regulatory body	

## Results

In the College of Medicine, King Saud University, Riyadh, Saudi Arabia there are approximately 1431 medical students enrolled, from the first year to final year medical degree program. Out of a total 1431 students, 750 medical students provided consent to participate in the study and 50 faculty members from basic and clinical departments of the College of Medicine were randomly selected for this study (Table 2).

**Table 2:** Participant demographic data

Sl. No	Demographic question	Students				Faculty		
1	Do you wish to participate in the survey?	Yes		No		Yes	No	
		750 (99.60 %)		3 (0.40)		50 (100 %)	0	
2	What is your gender?	Female	Male	Prefer not to say		Female	Male	
		311 (40.97 %)	441 (58.57 %)	1 (0.13 %)		18 (36 %)	32 (64 %)	
4	Please select a) Area of study (for students) b) Area of teaching (for faculty)	Medicine 753 (100 %)				Doctors (Clinical)	Doctors (Non-clinical)	Others
						21 (42 %)	25 (50 %)	4 (8 %)
5	Which year of your course are you currently in?	1st year	2nd year	3rd year	4th year	5th year	---	
		162 (21.5 %)	195 (25.9 %)	160 (21.2 %)	114 (15.1 %)	122 (16.2 %)		

Table 3 indicates 17 behaviors (50 % of the total) where none of the faculty respondents recommended Ignore while students showed a variable Ignore sanctions in a range of 6–29 %, e.g. *Signing attendance sheets for absent friends, or asking classmates to sign attendance sheets for you in labs or lectures (29.01 %), Resubmitting work previously submitted for a separate assignment or earlier degree (24.46 %), Lack of punctuality for classes (19.81 %), Inventing extraneous circumstances to delay sitting an exam (15.15 %), Not doing the part assigned in group work (14.29 %), Removing an assigned reference from a shelf in the library in order to prevent other students from gaining access to the information in it (11.88 %), intentionally falcifying results or treatment records in order to disguise mistakes(9.73 %), Posting inappropriate material about fellow students, teachers or patients on social media (9.50 %), Attempting to use personal relationships, bribes or threats to gain academic advantages by e.g. getting advance copies of exam papers or passing exam by such pressures on staff (9.33 %), Involvement in paedophilic activities - possession/viewing of child pornography images or molesting children (9.09 %), Drinking alcohol over lunch and interviewing a patient in the afternoon (8.57 %), Engaging in substance misuse e.g. drugs (7.48 %), Providing illegal drugs to fellow students (6.94 %), Cheating in an exam by e.g. copying from neighbour, taking in crib material or using mobile phone or getting someone else to sit for you (6.68 %), Threatening or verbally abusing a university employee or fellow student (6.54 %), Physically assaulting a university employee or student (6.53 %), Sexually harassing a university employee or fellow student (6.41 %).*

**Table 3:** Faculty and students differences in recommending ignore sanction for unprofessional behaviors

S No	Survey statement	Faculty *n* = 50	Students *n* = 750
1	Getting or giving help for course work against a teacher’s rule (e.g. Lending work to another student to look at)	4.00 %	23.73 %
2	Removing an assigned reference from a shelf in the library in order to prevent other students from gaining access to the information in it	0 %	11.88 %
3	Signing attendance sheets for absent friends, or asking classmates to sign attendance sheets for you in labs or lectures	0 %	29.01 %
4	Drinking alcohol over lunch and interviewing a patient in the afternoon	0 %	8.57 %
5	Exchanging information about an exam before it has been taken (e.g. OSCE)	24.00 %	41.42 %
6	Forging a healthcare worker’s signature on a piece of work, patient chart, grade sheet or attendance form	4.00 %	9.32 %
7	Claiming collaborative work as one’s individual effort	8.00 %	14.29 %
8	Altering or manipulating data (e.g. Adjusting data to obtain a significant result)	6.00 %	10.80 %
9	Failure to follow proper infection control procedures	4.00 %	9.36 %
10	Threatening or verbally abusing a university employee or fellow student	0 %	6.54 %
11	Attempting to use personal relationships, bribes or threats to gain academic advantages by getting advance copies of exam papers or passing an exam by such pressures on staff	0 %	9.33 %
12	Engaging in substance misuse (e.g. Drugs)	0 %	7.48 %
13	Completing work for another student	4.08 %	28.00 %
14	Intentionally falsifying results or treatment records in order to disguise mistakes	0 %	9.73 %
15	Physically assaulting a university employee or student	0 %	6.53 %
16	Purchasing work from a fellow student or internet, etc. supplier	12.24 %	18.21 %
17	Lack of punctuality for classes	0 %	19.81 %
18	Providing illegal drugs to fellow students	0 %	6.94 %
19	Not doing the part assigned in group work	0 %	14.29 %
20	Examining patients without knowledge or consent of supervising clinician	2.00 %	20.27 %
21	Sabotaging another student’s work	6.12 %	8.13 %
22	Inventing extraneous circumstances to delay sitting an exam	0 %	15.15 %
23	Sexually harassing a university employee or fellow student	0 %	6.41 %
24	Resubmitting work previously submitted for a separate assignment or degree	0 %	24.46 %
25	Plagiarizing work from a fellow student or publications/internet	4.08 %	12.20 %
26	Cheating in an exam by e.g. copying from neighbour, taking in crib material or using a mobile phone or getting someone else to sit for you	0 %	6.68 %
27	Cutting and pasting or paraphrasing material without acknowledging the source	2.00 %	14.29 %
28	Damaging public property, e.g. scribbling on desks or chairs	2.00 %	9.33 %
29	Falsifying references or grades on curriculum vitae or altering grades in the official record	2.04 %	9.12 %
30	Involvement in pedophilic activities - possession/viewing of child pornography images or molesting children	0 %	9.09 %
31	Photographing dissection or pro-section or cadaver materials	38.00 %	29.60 %
32	Joking or speaking disrespectfully about bodies/body parts	2.00 %	21.93 %
33	Inappropriate representation of Medicine in social media by posting photos/videos/texts about class or clinic activities	2.00 %	20.21 %
34	Posting inappropriate material about fellow students, teachers or patients on social media	0 %	9.50 %

Notably, there was a sevenfold increase in ignore sanction was observed with students (28.00 %) in comparison with faculty (4.08 %) for *completing work for another student*. On the other hand, there was a higher Ignore recommendation from faculty, i.e. (38.00 %) when compared with students (29.60 %) for *photographing dissection or prosection or cadaver materials.*

Tables 4 and 5 shows the median recommended sanctions by respondents (faculty and students) to 34 lapses in poly-professionalism in the undergraduate medical students at College of Medicine, King Saud University. The important findings are as follows:

**Table 4:** Recommended responses by median to 34 lapses in poly-professionalism in undergraduate students by faculty and medical students

S No	Survey statement	Faculty	Students
1	Getting or giving help for course work against a teacher’s rules (e.g. Lending work to another student to look at)	2	3
2	Removing an assigned reference from a shelf in the library in order to prevent other students from gaining access to the information in it	4	4
3	Signing attendance sheets for absent friends, or asking classmates to sign attendance sheets for you in labs or lectures	3.5	2
4	Drinking alcohol over lunch and interviewing a patient in the afternoon	6	6
5	Exchanging information about an exam before it has been taken (e.g. OSCE)	3	1
6	Forging a healthcare worker’s signature on a piece of work, patient chart, grade sheet or attendance form	6	5
7	Claiming collaborative work as one’s individual effort	5	4
8	Altering or manipulating data (e.g. Adjusting data to obtain a significant result)	6	4
9	Failure to follow proper infection control procedures	4.5	4
10	Threatening or verbally abusing a university employee or fellow student	5.5	5
11	Attempting to use personal relationships, bribes or threats to gain academic advantages by getting advance copies of exam papers or passing exam by such pressures on staff	7	5
12	Engaging in substance misuse (e.g. Drugs)	6	5
13	Completing work for another student	5	3
14	Intentionally falsifying results or treatment records in order to disguise mistakes	5	5
15	Physically assaulting a university employee or student	6.5	5
16	Purchasing work from a fellow student or internet, etc. supplier	5	4
17	Lack of punctuality for classes	3	2
18	Providing illegal drugs to fellow students	9	7
19	Not doing the part assigned in group work	4	3
20	Examining patients without knowledge or consent of supervising clinician	4	3
21	Sabotaging another student’s work	5	5
22	Inventing extraneous circumstances to delay sitting an exam	4	3
23	Sexually harassing a university employee or fellow student	8	7
24	Resubmitting work previously submitted for a separate assignment or degree	5	3
25	Plagiarizing work from a fellow student or publications/internet	6	4
26	Cheating in an exam by e.g. copying from neighbour, taking in crib material or using mobile phone or getting someone else to sit for you	6	5
27	Cutting and pasting or paraphrasing material without acknowledging the source	4	3
28	Damaging public property, e.g. scribbling on desks or chairs	4	4
29	Falsifying references or grades on curriculum vitae or altering grades in the official record	7	5
30	Involvement in pedophilic activities - possession/viewing of child pornography images or molesting children	9	7
31	Photographing dissection or pro-section or cadaver materials	2.5	3
32	Joking or speaking disrespectfully about bodies/body parts	4	2
33	Inappropriate representation of Medicine in social media by posting photos/videos/texts about class or clinic activities	4	2
34	Posting inappropriate material about fellow students, teachers or patients on social media	6	4

**Table 5:** Two level difference between the sanctions approved by faculty and students in the recommended sanctions for 12 lapses (35 % of the total)

S No	Survey statement	Faculty	Students	Chi-Square (*p*-value)
1	Exchanging information about an exam before it has been taken (e.g. OSCE)	3	1	60.00 (0.050)
2	Altering or manipulating data (e.g. Adjusting data to obtain a significant result)	6	4	
3	Attempting to use personal relationships, bribes or threats to gain academic advantages by getting advance copies of exam papers or passing exam by such pressures on staff	7	5	
4	Completing work for another student	5	3	
5	Providing illegal drugs to fellow students	9	7	
6	Resubmitting work previously submitted for a separate assignment or degree	5	3	
7	Plagiarizing work from a fellow student or publications/internet	6	4	
8	Falsifying references or grades on curriculum vitae or altering grades in the official record	7	5	
9	Involvement in pedophilic activities - possession/viewing of child pornography images or molesting children	9	7	
10	Joking or speaking disrespectfully about bodies/body parts	4	2	
11	Inappropriate representation of Medicine in social media by posting photos/videos/texts about class or clinic activities	4	2	
12	Posting inappropriate material about fellow students, teachers or patients on social media	6	4	

The overall difference between faculty and students was highly statistically significant (*p* < 0.050)

Faculty and students recommended similar sanctions for the following 5 lapses (14.7 % of the total) in professional behaviors (1) *Removing an assigned reference from a shelf in the library in order to prevent other students from gaining access to the information in it*, sanction = Reprimand, plus mandatory counselling (2) *Damaging public property e.g. scribbling on desks or chairs,* sanction = Reprimand, plus mandatory counselling (3) *Intentionally falsifying test results or treatment records in order to disguise mistakes*, sanction = Reprimand, counselling, extra work assignment (4*) Sabotaging another student's work*, sanction = Reprimand, counselling, extra work assignment (5) *Drinking alcohol over lunch and interviewing a patient in the afternoon*, here the median recommendation sanction was “Failure of specific class/remedial work to gain credit”.

Faculty recommended much higher sanction than the students for two behaviours (1) *Providing illegal drugs to fellow students* and (2) *Involvement in paedophilic activities - possession/viewing of child pornography images or molesting children*, faculty response was strict as recommended a median sanction of “Expulsion from college (no chance for readmission)”, while the students’ response was “Failure of specific year (repetition allowed). Furthermore, when asked about *Joking or speaking disrespectfully about bodies/body parts by students* as first time lapse in professional behavior, faculty members recommended median sanctions was “Reprimand, plus mandatory counseling”, while students approved “Reprimand (verbal warning)”.

## Discussion

Lapses in academic integrity are almost a universal concern according to a number of researchers. The period of study at the medical school is the foundation stone for ethical and moral value carried by future physicians of the society. According to Papadakis et al. [10, 11] medical students demonstrated unprofessional behaviour in medical school were more likely to have a subsequent state board disciplinary action. According to Ryan et al. [12] professionalism assessments addressed cognitive and behavioural outcomes. Roff et al. [9] surveyed using the same inventory and reported that 54 % of the students recommended sanctions in Scottish medical school in relation to lapses in academic integrity. Similarly, Shukr [13] and Babely et al. [14] also used same inventory and identified serious issues related to academic integrity that require identification and their solution. The revelations of our study were consistent with the above findings. Our study demonstrated that the cheating is not confined to examination such as copying from neighbour, taking in crib material or using mobile phone or getting someone else to sit for you, but also included lapses in academic integrity such as Signing attendance sheets for absent friends, or asking classmates to sign attendance sheets for you in labs or lectures and Resubmitting work previously submitted for a separate assignment or earlier.

Some of the disclosures of this study were disturbing which warrant an immediate intervention by the ethical and regulatory authorities of the concerned departments. Data of the current result shows that the sanctions recommended by students of KSU were often much more lenient when compared to the faculty cohort of the same college. It was a disturbing observation where 29.01, 24.46 and 6.68 % of the students cohort recommended ‘Ignore’ sanction when compared to 0 % ‘Ignore’ from faculty cohort for unprofessional behaviours such as a) Signing attendance sheets for absent friends, or asking classmates to sign attendance sheets for you in labs or lecture, b) Resubmitting work previously submitted for a separate assignment or earlier degree and c) Cheating in an exam by e.g. copying from neighbour, taking in crib material or using mobile phone or getting someone else to sit for you, respectively. We found that for 27 lapses (79 % of the total) in professional behaviour sanctions recommended by students are lower when compared to faculty. Similar findings were reported that for some lapses the students recommended much lower sanctions as compared to the faculty [15].

Therefore, efforts are necessary to disseminate awareness on the importance of academic integrity among student community of the College of Medicine, KSU. As outlined above, the present study was an attempt to explore perceptions of participants from students and faculty cohorts of College of Medicine, KSU, Riyadh on lapses for professional behaviour by students in their college.

There were indications of alignment as well as differences in the responses of the respondents cohorts (in different countries). This reliable collection and comparison of the participants’ perception of medical professionalism enabled us to determine prevalence of professionalism lapses related to academic integrity by students in college of medicine, KSU. It indicates an urgent need to improve certain areas of professional teaching activity. Hence it is imperative to promote and disseminate the values of academic integrity and professional behaviours by enhanced teaching methods and implement certain regulations on polyprofessionalism in order to help the students to prepare for their responsibilities as future practicing doctors.

The present study helps to determine the prevalence of professionalism lapses related to academic integrity by students in College of Medicine, King Saud University, Riyadh. This information in turn can be used to target further education in expected standards of professionalism. It also enables College of Medicine, KSU curriculum planners to identify where intervention regarding teaching of professionalism is required.

The present study has been limited to testing the feasibility of an online inventory to ‘map’ student and faculty understanding of the relative importance of various lapses in academic integrity through the ‘proxy’ of soliciting recommended sanctions. We suggest that mapping existing norms, as well as those of patients and public may help students and the profession move closer towards an “ideally” defined state of professional conduct. Respondence rates among the 52 % of the target propularion ranged from 26 to 15 % from the different year groups and the analysis of the year differences will be the subject of a separate paper.

## Conclusions

The Dundee Polyprofessionalism Inventory-1 is a useful tool to measure and diagnose the professionalism related to academic integrity at undergraduate level. There are notable issues related to academic integrity in the students at College of Medicine, KSU, as they prefer to opt for the Ignore sanction which amount to serious lapses in academic integrity. This necessitates urgent intervention by the teaching fraternity to help students understand the impact of such perception of professional integrity.
